# The “Connection” Between HIV Drug Resistance and RNase H

**DOI:** 10.3390/v2071476

**Published:** 2010-07-21

**Authors:** Krista A. Delviks-Frankenberry, Galina N. Nikolenko, Vinay K. Pathak

**Affiliations:** Viral Mutation Section, HIV Drug Resistance Program, National Cancer Institute at Frederick, Frederick, Maryland 21702, USA; E-Mails: frankenk@mail.nih.gov (K.A.D.-F.); gnikolenko@comcast.net (G.N.N.)

**Keywords:** connection subdomain, NRTI, NNRTI, RNase H, drug resistance, HIV

## Abstract

Currently, nucleoside reverse transcriptase inhibitors (NRTIs) and nonnucleoside reverse transcriptase inhibitors (NNRTIs) are two classes of antiretroviral agents that are approved for treatment of HIV-1 infection. Since both NRTIs and NNRTIs target the polymerase (pol) domain of reverse transcriptase (RT), most genotypic analysis for drug resistance is limited to the first ∼300 amino acids of RT. However, recent studies have demonstrated that mutations in the C-terminal domain of RT, specifically the connection subdomain and RNase H domain, can also increase resistance to both NRTIs and NNRTIs. In this review we will present the potential mechanisms by which mutations in the C-terminal domain of RT influence NRTI and NNRTI susceptibility, summarize the prevalence of the mutations in these regions of RT identified to date, and discuss their importance to clinical drug resistance.

## 1. Introduction

The first cases of acquired immunodeficiency syndrome (AIDS) and HIV-1 infection were reported in the early 1980s [1–3]. Today, over 33 million people world-wide are currently infected with the virus, with a reported two million individuals dying of the disease over the last year (http://www.unaids.org). The advent of highly active antiretroviral therapy (HAART) has decreased the mortality rate of HIV-1 infected patients and helped to significantly extend the lifespan of people living with AIDS. Drugs used to treat HIV-1 target essential enzymes in the life cycle of the virus, namely protease, reverse transcriptase (RT) and integrase, as well as proteins essential for viral fusion and entry into the target cell. One of the major impediments to HIV-1 therapy is the rapid accumulation of mutations that arise within the virus which overcome the effectiveness of the drugs. Unfortunately, this inability to control the replication of the virus eventually leads to virologic failure and progression to AIDS.

The first drug approved for treatment of HIV-1 infected patients was AZT – a drug targeted to the HIV-1 RT [4]. RT is composed of two subunits: the p66 subunit contains the polymerase (pol) domain, the connection (cn) subdomain, and the RNase H (rh) domain, while the p51 subunit is a proteolytically cleaved product of the p66 subunit lacking the rh domain and polymerase activity [5,6]. Together, these proteins fold independently to function as a p66/p51 heterodimer during reverse transcription, creating a double-stranded DNA copy of the viral RNA genome, while also degrading the original viral RNA template (for an overview of reverse transcription, see ref [7]).

Currently, all drugs approved by the United States Food and Drug Administration against RT target the polymerase active site or a drug-binding site near the active site in the pol domain. Therefore, most commercial genotypic assays analyze the first ∼300 amino acids of RT and identify mutations located in this region for use in guiding patient drug regimens [8,9]. It has been argued that this is a reasonable compromise between the cost of drug resistance testing and maximizing clinically useful information. However, recent data has emerged to suggest that mutations that lie outside of the pol domain, within the C-terminal domain of RT (amino acids 312–560), can significantly increase resistance to nucleoside as well as non-nucleoside RT inhibitors. These findings indicate that standard genotypic and phenotypic analyses of drug resistance should include the C-terminal domains of RT. In this review, we will discuss biochemical mechanisms by which these mutations influence drug susceptibility and analyze the contributions of mutations in the C-terminal domain to clinical drug resistance.

## 2. Reverse Transcriptase Inhibitors and Drug Resistance Mutations in the pol Domain

Drugs targeted to RT fall into two classes: nucleoside RT inhibitors (NRTIs) and non-nucleoside RT inhibitors (NNRTIs). Currently there are eight NRTIs (abacavir [ABC], zidovudine [AZT], zalcitabine [ddC], didanosine [ddI], stavudine [d4T], emtricitabine [FTC], lamivudine [3TC], and tenofovir disoproxil fumarate [TDF]) and four NNRTIs (delaviridine [DLV], efavirenz [EFV], etravirine [ETR], and nevirapine [NVP]) approved for use in treatment of HIV-1 infection. NRTIs are nucleoside analogs that lack the 3′OH on the sugar ring and competitively block reverse transcription by causing chain termination during DNA polymerization [10,11]. NRTIs are prodrugs that require intracellular phosphorylation to the 5′-triphosphate form by host cell kinases in order to become active. On the other hand, NNRTIs in general are non-competitive inhibitors of RT that bind to a hydrophobic pocket near the polymerase active site, inducing conformational changes that inhibit RT enzymatic activity [12]. As expected, treatment with either or both classes of drugs causes the emergence of drug resistance mutations generally clustered around the NRTI or NNRTI binding sites.

Patients treated with NRTIs develop classical patterns of resistance-associated mutations in the pol domain [13]. K65R characteristically arises with ABC, ddI, FTC, 3TC or TDF treatment, while M184V arises with ABC, FTC or 3TC treatment. K70R is common to d4T, TDF, and AZT therapy, while L74V arises in response to ABC and ddI treatment. Typically, thymidine analog mutations (TAMs) arise with AZT and d4T treatment, which encompass M41L, D67N, K70R, L210W, T215F/Y, and K219Q/E/N. Different patterns of TAMs accumulate in patients, which segregate into two distinct pathways named TAM-1 and TAM-2 [14–16]. The TAM-1 pathway includes M41L, L210W and T215Y, whereas the TAM-2 pathway includes D67N, K70R, T215F and K219Q/E/N. The cumulative addition of TAMs results in high levels of NRTI resistance, and which pathway predominates is likely driven by the first mutation acquired in the patient [15]. In addition to the above described primary NRTI mutations, 61 novel accessory mutations in the pol domain have also been described to influence NRTI resistance (reviewed in [17]). These accessory mutations usually enhance resistance in the presence of classical NRTI mutations and/or enhance the replicative capacity of the virus.

As for NNRTIs, classical resistance-associated mutations in the pol domain are also selected in response to NNRTI therapy. Patients on EFV and NVP treatment typically acquire mutations L100I, K103N, V106A/M, V108I, Y181C/I, Y188C/H/L and G190A/S [13]. After treatment with ETR [18], the newly approved NNRTI, common mutations that are selected include L100I, K101E/H/P and Y181C/I/V [13]. Fortunately, K103N so far has not been associated with ETR resistance, and therefore ETR appears to be a good NNRTI for salvage therapy. As with NRTIs, 33 NNRTI accessory mutations in the pol domain have also been identified from patient databases to be associated with NNRTI therapy including K101Q, I135T/M and L228H/R (reviewed in [17]). Closer examination of the accessory mutations is needed to assess their role in influencing the evolution of drug resistance in patients.

## 3. Mechanisms of NRTI Resistance Associated with the pol Domain

**Figure 1:**
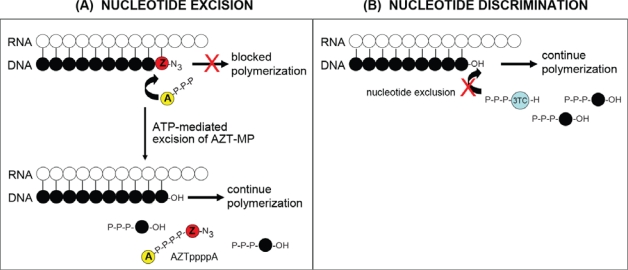
Mechanisms of NRTI resistance. **(A)** Nucleotide excision. Mutations in pol, such as TAMs, aid in the ATP-mediated removal of an incorporated AZT monophosphate (AZT-MP) yielding an AZTppppA excision byproduct. **(B)** Nucleotide discrimination. Mutations in pol cause steric hindrance at the pol active site, excluding certain drugs, for example 3TC, from being incorporated during reverse transcription. Both examples yield a complex competent for polymerization. Yellow circle with the letter A and three phosphates, ATP; black circles with three phosphates, dNTPs; red circle with the letter Z and the N3 azido group, AZT-MP; blue circle with three phosphates, 3TC-triphosphate; P, phosphate group. RNA is depicted with white circles; DNA is depicted with black circles.

Understanding how HIV-1 RT can overcome inhibition by antiviral drugs to successfully complete reverse transcription is crucial for clinical management of HIV-1 infection and for improving the efficacy of new drugs. Analysis of pol resistance mutations from patient viruses has established two main mechanisms by which HIV-RT exhibits a NRTI-resistant phenotype. These two mechanisms, namely nucleotide excision and nucleotide discrimination, are briefly outlined in Figure 1. As shown in Figure 1A, an NRTI is incorporated into nascent DNA by RT, and the absence of a 3′ OH group results in termination of DNA synthesis. RT lacks 3′-5′ proofreading activity, but nevertheless can remove the incorporated NRTI by reversing the polymerization step; although inorganic pyrophosphate and ATP can both act as a pyrophosphate donor *in vitro*, ATP is the likely substrate that is used in cells to excise AZT-monophosphate (AZT-MP) by forming a dinucleoside tetraphoshate (AZTppppA). Nucleotide excision catalyzed by wild-type RT is inefficient, but the presence of TAMs enhances the binding/placement of ATP in the RT active site [19]. Several studies have shown that AZT-resistant virus carrying TAMs were more efficient at unblocking AZT-MP terminated primers than wild-type RT when ATP was used as the pyrophosphate donor [19–25]. It has also been shown that other nucleoside triphosphates can act as pyrophosphate donors; however it is likely that ATP serves as the main donor for excision in macrophages and unstimulated T cells [24].

Observations that some NRTI-resistant viruses were able to selectively reduce the incorporation of the inhibitor lead to a second mechanism of NRTI inhibition known as nucleotide discrimination (Figure 1B). Most of the mutations that are involved with nucleotide discrimination, such as M184V, K65R, L74V and Q151M, affect critical residues in the nucleotide binding site of pol that are important for interacting with the incoming dNTP. M184V/I is a classic example of nucleotide discrimination, with crystal structure analysis [26] revealing that these mutations create steric hindrance between the oxathiolane ring of the 3TC-triphosphate and the side chain of the beta-branched amino acids at position 184, reducing the incorporation of 3TC-triphosphate [27–29]. Nucleotide excision is the primary mechanism by which HIV-1 acquires resistance to AZT and d4T, whereas nucleotide discrimination is the primary mechanism by which resistance to 3TC and FTC is acquired. For a more thorough discussion of these mechanisms, please refer to excellent reviews in this and other issues of *Viruses* by Acosta-Hoyas *et al.* [30] and Singh *et al.* [31].

## 4. Mechanisms of NRTI Resistance Associated with the cn and rh Domains

Studies performed by several groups over the last five years have established a role for mutations in the cn and rh domains in NRTI resistance. Furthermore, these studies have begun to provide valuable insights into the mechanisms by which these mutations, which are generally over 30 angstroms away from the pol active site, increase resistance to NRTIs. The current understanding of these mechanisms of NRTI resistance is discussed below.

### 4.1. RNase H-dependent Mechanism for NRTI resistance: Balance between Nucleotide Excision and RNase H Activity

**Figure 2:**
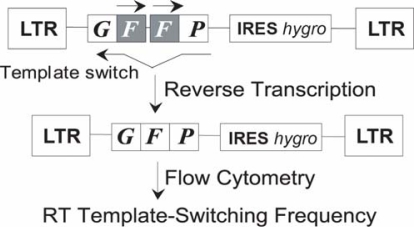
Single-cycle direct repeat deletion assay to determine the percentage of template switching *in vivo*. Proviruses containing a direct repeat (horizontal arrows) of the green fluorescent protein gene (GFP) were mobilized and used to infect target cells. The frequency of a homologous template switch during reverse transcription in target cells, which reconstitutes a functional GFP gene, was measured by flow cytometry. IRES, internal ribosomal entry site; *hygro*, hygromycin gene; LTR, long terminal repeats.

A third mechanism for NRTI drug resistance was proposed by Nikolenko *et al.*, in which mutations that reduce RNase H cleavage can contribute to the NRTI-resistant phenotype by providing more time for RT to carry out nucleotide excision and resume productive DNA synthesis [32–34]. This proposal was based on the observation that AZT treatment results in an increase in RT template switching events during viral replication. A previously described template switching assay is outlined in Figure 2 [32]. In this assay, an HIV-1 vector that contains direct repeats of the middle portion of the GFP gene (the “F” portion) is mobilized by transfection with HIV-1 Gag-Pol and envelope expression plasmids. An RT template switch within the F portion of GFP results in functional reconstitution of GFP in a single cycle of viral replication. It has been previously shown that reducing RNase H cleavage decreases RT template switching, whereas slowing down DNA synthesis increases RT template switching [32,35]. It was observed that reverse transcription in the presence of AZT increased the rate of RT template switching in a dose-dependent manner [35]. From this result, it was postulated that AZT-MP incorporation in the nascent DNA results in a stalled reverse transcription complex; however, at a certain rate, wild-type RT excises the incorporated AZT-MP and resumes DNA synthesis. The AZT-MP incorporation and excision slows down DNA synthesis, which in turn increases the rate of RT template switching.

**Figure 3:**
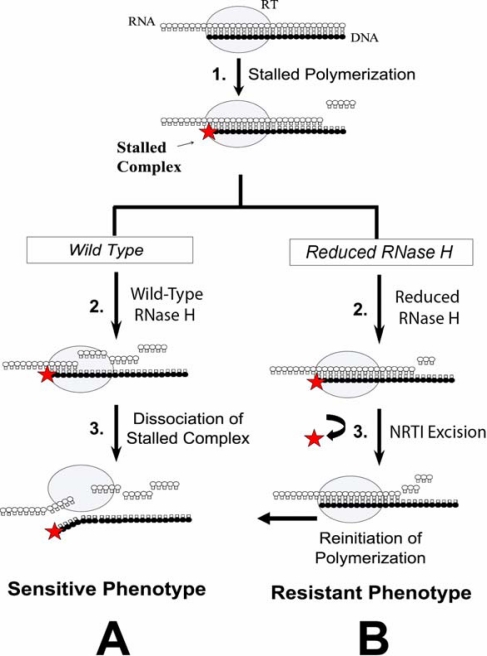
Mechanisms of C-terminal domain NRTI resistance. During reverse transcription, incorporation of AZT leads to a complex stalled for polymerization. **(A)** In the case of a wild-type RT with wild-type RNase H activity, the stalled complex leads to a dissociation of the complex and sensitive phenotype as RNase H cleavage causes minimal stretches of homology to be retained between the RNA/DNA hybrid. **(B)** In the case of an RT with reduced RNase H activity, the decrease in template RNase H cleavage allows longer stretches of homology to be retained between the RNA/DNA hybrid giving more time for the pol active site to undergo nucleotide excision and reinitiate polymerization, leading to a resistant phenotype. Gray oval, reverse transcriptase; star, AZT; white circles, RNA; black circles, DNA.

These observations led to a model in which reducing RNase H cleavage allows RT more time to excise AZT-MP, which results in a higher level of AZT resistance. As shown in Figure 3, the model proposes that AZT-terminated reactions will form stalled complexes that are polymerization incompetent. Under normal conditions and wild-type RNase H activity, this stalled complex will dissociate and terminate reverse transcription (Figure 3A). However, as shown in Figure 3B, RTs carrying mutations that reduce RNase H cleavage will create longer stretches of homology between the RNA template and the DNA primer strands during reverse transcription, providing more time for the polymerase to carry out excision of the incorporated AZT-MP, leading to the resumption of DNA synthesis and a resistant phenotype. This prediction was confirmed by the observation that H539N and D549N mutations, which are near the RNase H active site and reduce RNase H activity [35–38], conferred high levels of AZT and d4T resistance [35]. This increase in AZT resistance was synergistic with TAMs; while the TAMs increased AZT resistance 23-fold, addition of D549N to the TAMs increased AZT resistance ∼1250-fold, relative to wild-type RT. These results demonstrated that the dynamic steady-state between the polymerase and RNase H activities was an important determinant of NRTI resistance.

To explore the clinical relevance of this mechanism of resistance, Nikolenko *et al.* determined whether the C-terminal domains of RTs derived from treated patients contained mutations that increase NRTI resistance [33]. Analysis of the C-terminal domains of seven treatment-experienced patients showed that the patient-derived cn subdomains increased AZT resistance by as much as a 536-fold in the context of TAMs. Mutational analysis of these cn subdomains resulted in the identification of eight novel mutations, E312Q, G335C/D, N348I, A360I/V, V365I, and A376S, that significantly contributed to AZT resistance. The results also showed that the patient cn subdomains decreased template switching, which is consistent with the prediction that these mutations reduce RNase H activity [33].

Brehm and colleagues sought to explore the role of mutations in the C-terminal domains of RT in AZT resistance by selecting for AZT-resistant variants in cell culture [39]. In the course of *in vitro* passaging experiments, they found that in addition to TAMs, they selected for A371V in the cn subdomain and Q509L in the RNase H domain of RT. Mutational analysis confirmed that these mutations increased AZT resistance in the context of TAMs 10–50-fold, but had little effect in the absence of TAMs.

Mutations in the C-terminal domains may reduce RNase H activity by directly affecting the RNase H cleavage activity of RNase H, or indirectly by affecting the positioning of the template-primer substrate at the RNase H active site. Several amino acids in the cn subdomain and RNase H domain of RT contact the primer strand and form an RNase H primer grip structure [40–45], which helps to properly position the RNA-DNA hybrid at the RNase H active site to facilitate efficient RNA cleavage. Point mutation studies on the RNase H primer grip have shown that several functions of RT are affected including deficient DNA synthesis, reduced RNase H activity, poor PPT cleavage and/or reduced strand transfer efficiency [46–50]. Furthermore, in murine leukemia virus, mutation Y586F in the RT RNase H primer grip (equivalent to Y501F in HIV-1), has been shown to be important for the overall fidelity of DNA synthesis and the proper positioning of the RNA/DNA hybrid at the both the polymerase and RNase H active site [51]. Delviks-Frankenberry *et al.* hypothesized that the mutations in the cn subdomain that increase AZT resistance do so by affecting the RNase H primer grip, which results in decreased RNase H activity. To explore this aspect of the model, the effects of alanine substitutions at RNase H primer grip residues on AZT resistance were determined [52]. The results showed that 10 of the 11 substitution mutations (G359A, A360K, K390A, K395A, E396A, T473M, Q475A, K476A, Y501A and I505A) increased AZT resistance and decreased RT template switching, again supporting the idea that increases in NRTI resistance are related to decreases in RNA template degradation. Overall, these data further supported the authors’ previous hypothesis and showed that cn mutations may affect the positioning of the RNase H primer grip amino acids, leading to a repositioning of the template-primer at the RNase H active site and thereby reducing RNase H activity [33,35].

Key predictions of this model were further tested by Delviks-Frankenberry and colleagues by carrying out detailed biochemical analysis of the cn subdomains of RTs derived from treatment-experienced patients. The results showed that the cn subdomains from treatment-experienced patients (in the context of TAMs) decreased primary and secondary RNase H cleavages and enhanced ATP-mediated AZT-MP excision on an RNA template, but not a DNA template [34,53]. Furthermore, the reductions in RNase H activity were attributed to the eight specific cn subdomain mutations (E312Q, G335C/D, N348I, A360I/V, V365I, and A376S) that were primarily responsible for the increase in NRTI resistance [33].

The studies by Yap and colleagues also confirmed and supported this model by examining in depth the cn mutation N348I. They found that N348I does not enhance NRTI resistance by discrimination, but instead functions to reduce secondary RNase H cleavages and enhances ATP-mediated AZT-MP excision only on an RNA template [54]. Furthermore, a recent analysis by Radzio *et al.* examined the how mutations such as Y181C, L74V or M184V which are antagonistic to the TAMs mutations could be selected together in the same virus [55]. They found that N348I, which has reduced RNase H cleavage, compensated for the reduced ATP-mediated AZT excision associated with Y181C, L74V or M184V on an RNA/DNA template, but not a DNA/DNA template, demonstrating an RNase H-dependent mechanism for selecting antagonistic mutations in the virus.

Overall, these studies support an RNase H-dependent mechanism for cn mutations contributing to enhanced NRTI excision and resistance. The cn subdomain mutations increase NRTI resistance synergistically with TAMs. In the absence of TAMs, the cn mutations generally have a significantly reduced impact on NRTI resistance (less than two-fold) in single-cycle assays performed in cell culture. Nevertheless, these relatively small increases in NRTI resistance are likely to be biologically significant, because the cn mutations appear to be selected early in treatment and often appear before TAMs. The cn mutations appear to increase NRTI resistance in the absence of TAMs *in vivo* by affecting the balance between a low level of NRTI excision exhibited by wild-type RT and RNase H activity. Thus, selection of cn mutations early in treatment over the course of multiple cycles of replication may contribute to the subsequent selection of TAMs.

### 4.2. RNase H-independent Mechanisms of NRTI Resistance

In addition to reducing template RNA degradation, cn mutations may also directly improve the ability of RT to carry out nucleotide excision. The hypothesis that cn mutations directly increase NRTI resistance through an RNase H-independent mechanism was recently explored in biochemical analyses of RT mutants [34,56–58]. Zelina *et al.* analyzed the effect of cn subdomain mutation G333D in the presence of TAMs and/or M184V on AZT and 3TC resistance [56]. It is known that M184V reverses the effects of TAMs and restores AZT sensitivity [59–62]; the authors found that in the presence of TAMs and M184V, G333D increased the ability of the RT to bind to the template-primer, and increased ATP-mediated excision. In addition, G333D increased discrimination against 3TC incorporation in the presence of M184V. The G333D mutation did not exhibit reductions in RNase H cleavage, and it was concluded that in the context of TAMs and M184V, G333D directly affects the polymerase active site, presumably as a result of long-range interactions and conformational changes in the cn subdomain.

In a recent study by von Wyl *et al.*, the relationship between M184V and N348I was examined [63]. They showed that M184V not only has reduced DNA polymerization, but also reduced PPi-mediated AZT excision and reduced ATP-mediated excision in the presence of TAMs. However, the addition of cn mutation N348I compensated for these defects suggesting that the cn mutations could also counteract enzymatic defects introduced in the pol domain (M184V). Whether this compensation was attributed to N348I’s reduced RNase H cleavage needs further examination. Nevertheless, this study shows how mutations selected by two different drugs (M184V for 3TC and N348I for AZT) can be selected together in the same virus to gain a replicative advantage in the presence of drug pressure.

Ehteshami and colleagues [58] analyzed the effects of N348I and A360V mutations in combination with TAMs on AZT-MP excision using an RNA/DNA hybrid substrate. They found that these mutations enhanced AZT-MP excision even in the presence of an RNase H-inactivating E478Q mutation, indicating that in addition to an RNase H-dependent mechanism, an RNase H-independent mechanism also contributes to the increase in AZT resistance. They also found that the cn subdomain mutations increased the processivity of RT, which could account for more efficient AZT-MP excision.

Delviks-Frankenberry and colleagues [34] analyzed the effects of cn subdomains of RTs derived from treatment-experienced patients and found that the cn subdomains increased ATP- and PPi-mediated AZT-MP excision on an RNA template but had minimal effects on AZT-MP excision on a DNA template; however, one of five cn subdomains did increase AZT-MP excision on an DNA template, and all showed a higher ratio of ATP- to PPi-mediated excision on both an RNA and DNA template. The differential effects on the use of ATP and PPi substrates suggested that in addition to an RNase H-dependent mechanism, there was a direct increase in AZT-MP excision at the polymerase active site where ATP and PPi bind.

Brehm and colleagues [57] have suggested a model in which the cn subdomain mutations cause RT to bind to the substrate RNA:DNA hybrid in a mode that favors nucleotide excision and disfavors RNase H cleavage. After primary RNase H cleavage, which reduces the RNA/DNA duplex to approximately 15–18 nucleotides, the RT dissociates and can reassociate with the duplex in either a polymerase-competent or an RNase H-competent mode. Analysis of Q509L and A371V/Q509L showed that the Q509L mutant prefers to bind in the polymerase-competent mode, which results in an increase in NRTI excision; the increased binding in the polymerase-competent mode reduces binding in the RNase H-competent mode, resulting in reduced RNase H cleavage. In contrast to Q509L, Ehteshami and colleagues found that binding in the polymerase-competent mode was not affected by the N348I mutation, and modestly increased by the A360V mutation [58]. Thus, different cn subdomain mutations may directly enhance nucleotide excision through different mechanisms.

Analysis of the cn subdomain of circulating recombinant form AE (CRF01_AE) provided additional evidence that mutations in this region can directly increase the efficiency of AZT-MP excision [64]. CRF01_AE containing TAMs exhibited a higher level of AZT resistance than subtype B containing the same TAMs (64-fold *vs.* 13-fold, compared to wild-type RT). The higher level of resistance was shown to be due to the T400 amino acid in the CRF01_AE cn subdomain. This amino acid is an alanine in subtype B, and an A400T substitution in subtype B increased AZT resistance, while a T400A substitution in CRF01_AE decreased AZT resistance. Interestingly, the A400T substitution in subtype B increased AZT-MP excision on both an RNA template and a DNA template, indicating that the increase in AZT resistance was likely to be a direct effect on nucleotide excision and not due to a decrease in RNase H activity.

Overall, these studies showed that cn subdomain mutations reduce RNase H activity and increase AZT resistance through both RNase H-dependent and RNase H-independent mechanisms.

## 5. Mechanisms of NNRTI Resistance Associated with the pol Domain

NNRTIs bind to a pocket in the palm subdomain of p66, between beta sheets β6-β10-β9 and β12-β13-β14 [65–67], likely distorting the position of the pol primer grip. This distortion of the pol primer grip in turn changes the positioning of the RNA/DNA template and/or conformation of the catalytic residues (YMDD motif) and inhibits DNA synthesis [68,69]. NNRTI resistance mutations generally alter interactions between the NNRTI and RT by affecting the affinity of the drug to the NNRTI-binding pocket [70–72]. Three basic mechanisms have been described for NNRTI resistance [70,71, 73,74]. First, NNRTI resistance mutations can disrupt specific contacts between the inhibitor at the entrance of the pocket. For example, K103N and K101E sit on the rim of the NNRTI binding pocket [70,75,76] blocking entry of the NNRTI. Second, NNRTI resistance mutations can disrupt important contacts in the interior of the NNRTI binding pocket. Y181C and Y188L lose important NNRTI aromatic ring interactions in the core of the NNRTI-binding pocket, decreasing binding of the NNRTI [65,77,78]. Third, NNRTI resistance mutations can change the global conformation or the size of the NNRTI-binding pocket. For example, G190E creates steric bulk in the β9–β10 hairpin of the pocket, leaving no room for the NNRTI to bind [66,79]. These different mechanisms of NNRTI resistance thus interfere with NNRTI binding to RT and allow reverse transcription to proceed.

## 6. Mechanisms of NNRTI Resistance Associated with the cn and rh Domains

Interestingly, some cn mutations, such as G335C, N348I, A360I/V, T369I/V, A376S, E399D and G333D/E not only increase resistance to NRTIs, but also NNRTIs [54, 80–86]. In the context of patient sequences, Nikolenko *et al.* showed that patient pol *vs.* pol + cn domains enhanced resistance to AZT [33] as well as NVP, DLV, EFV and ETR [86] for most patients. In addition, Gupta *et al.* showed that the addition of N348I or T369I to patient pol domains containing NRTI and/or NNRTI resistance mutations also enhanced resistance to not only AZT, but also DLV, EFV and NVP [85]. Since NRTIs and NNRTIs inhibit HIV-1 replication by different mechanisms, and mutations in the pol domain that confer resistance to these two drug classes generally do not to overlap, the mechanisms by which the cn subdomain mutations confer dual resistance are of great interest. Current studies that seek to elucidate these mechanisms are discussed below.

### 6.1. RNase H-Dependent Mechanism of NNRTI Resistance

**Figure 4:**
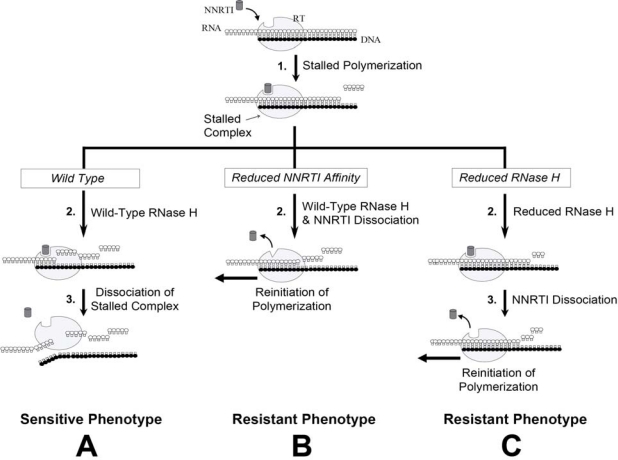
Mechanism of C-terminal domain NNRTI resistance. During reverse transcription, an NNRTI binds RT and forms a stalled complex. **(A)** In the case of a wild-type RT with wild-type RNase H activity, the stalled complex leads to a sensitive phenotype as RNase H cleavage causes minimal stretches of homology to be retained between the RNA/DNA hybrid. **(B)** In the case of RT mutations that reduce the affinity of the NNRTI for RT, the NNRTI has time to dissociate from the template-primer, forming a polymerization-competent complex and a resistant phenotype. **(C)** In the case of an RT with reduced RNase H activity, the reduction in template cleavage allows longer stretches of RNA/DNA hybrids to be retained, allowing more time for the NNRTI to dissociate and enable re-initiation of polymerization, leading to a resistant phenotype. Labels as in Figure 3; gray cylinder, NNRTI.

Nikolenko and colleagues have proposed that increases in NNRTI resistance observed with C-terminal domain mutations can also be explained by decreases in RNase H cleavage [86]. This model suggests a mechanism that is parallel to the RNase H-dependent mechanism of NRTI resistance outlined in Figure 3. When the NNRTI affinity to the wild-type RT is not affected by mutations and when the RNase H activity is wild-type, binding of the NNRTI to RT during reverse transcription forms a stalled complex, leading to dissociation of the reverse transcription complex and a sensitive phenotype (Figure 4A). As expected, NNRTI binding pocket mutants will decrease NNRTI binding to RT, and will increase NNRTI dissociation from the RT, leading to a resistant phenotype (Figure 4B). Mutations in cn and rh that reduce RNase H cleavage will allow more time for the NNRTI to dissociate from the NNRTI-RT-template/primer complex (NNRTI-RT-T/P), allowing the resumption of DNA synthesis and thereby resulting in enhanced NNRTI resistance (Figure 4C). In addition, combining mutations in RT that reduce NNRTI affinity with mutations in RT that reduce RNase H cleavage should further increase NNRTI resistance.

The RNase H-dependent NNRTI resistance model was tested by analyzing RNase H mutants D549N, Q475A and Y501A, which reduce RNA template degradation [86]. In each case, NVP and DLV resistance was enhanced, but EFV and ETR resistance was not altered. This correlated with the affinity of the NNRTIs to RT, and showed that NNRTIs such as EFV and ETR, which have a high affinity to RT, do not dissociate from the NNRTI-RT-T/P complex, even after RNase H activity is reduced. In other words, the time required for EFV and ETR to dissociate from the complex is longer than the time available before RNase H degradation results in dissociation of the complex and termination of reverse transcription.

To further test the RNase H-dependent NNRTI resistance model, Nikolenko *et al.* introduced mutations in the NNRTI binding pocket, which would be expected to reduce the affinity of EFV and ETR to the RT [86]. When they analyzed the effects of D549N in the presence of the NNRTI binding pocket mutations, they found that the RNase H mutation further increased EFV and ETR resistance. These results were consistent with the model that the high affinity of the EFV and ETR to the wild-type RT prevented the NNRTI-RT-T/P complex from being dissociated even after RNase H activity was reduced by the D549N mutation.

**Figure 5:**
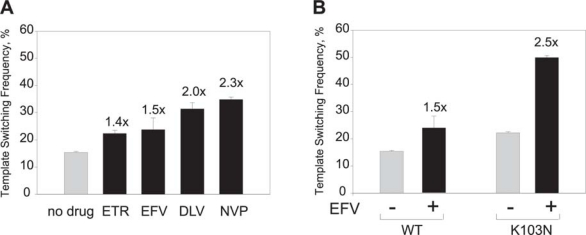
Effect of different NNRTIs on the frequency of RT template switching by wild-type and K103N mutant RTs **(A)** The effect of NNRTI treatment on the template switching frequency by wild type HIV-1 RT is dependent on the affinity of NNRTI to the RT. **(B)** Effect of decreased affinity of EFV to the drug resistant K103N mutant RT on the template switching frequency. Figure represents data described in [86].

A key component of this model is that NNRTI resistance is influenced by the interplay of NNRTI affinity to the RT and RNase H activity. Nikolenko *et al.* further explored this interplay *in vivo* by determining the effect of NNRTIs on RT template switching using the direct repeat deletion assay described in Figure 2. The RNase H-dependent NNRTI resistance model hypothesizes that NNRTIs establish a steady-state between the formation of a stalled NNRTI-RT-T/P complex and dissociation from the complex. Based on this model, NNRTIs will slow down DNA synthesis, leading to an increase in RT template switching frequency. The effect of each NNRTI on template switching is expected to be dependent on its affinity to the RT. Thus NNRTIs with high affinity to the RT (EFV and ETR) may form very stable NNRTI-RT-T/P polymerization incompetent complexes that will not resume DNA synthesis regardless of the RNase H template degradation rate; therefore, their effect on the template switching frequency should be minimal. As shown in Figure 5A, the increases in the template switching frequency for NVP, DLV, EFV, and ETR (2.3-, 2-, 1.5- and 1.4-fold, respectively) correlated with their IC_50_s (60 nM > 11 nM > 1.9 nM > 1.1 nM, respectively) and Kds (25 nM > 16.6 nM > 2 nM for NVP, DLV, and EFV, respectively) [86]).

Nikolenko and colleagues also tested the effect of EFV on the template switching frequency of the K103N mutant RT, which has reduced affinity to EFV [87–89], and found that at 95% inhibitory concentration, the template switching frequency was increased 2.5-fold for the K103N mutant compared to only 1.5-fold for wild-type RT (Figure 5B). This result is consistent with the view that reduced NNRTI affinity to the RT allows the establishment of a steady-state between the formation and dissociation of the NNRTI-RT-T/P complex, leading to more efficient resumption of DNA synthesis. Overall, these results support the role of RNase H as a unifying mechanism by which cn subdomain and rh domain mutations can exhibit dual NRTI and NNRTI resistance.

### 6.2. RNase H-independent Mechanisms of NNRTI Resistance Associated with C-terminal Domain Mutations

Proper heterodimerization of the HIV-1 RT p66 and p51 subunits is important for RT DNA polymerase and RNase H activities, and alteration of heterodimerization stability is likely to inhibit RT function (reviewed in [90–93]). It was observed that NNRTIs increased the stability of RT dimers [94], leading to the hypothesis that NNRTIs may inhibit HIV-1 replication by affecting heterodimer stability. Along these lines, Gupta, *et al.* observed that cn mutation T369I showed impaired gag processing and a decrease in p66/p51 dimerization, leading the authors to suggest that decreased dimerization could also lead to reduced RNase H activity [83]. If NNRTIs inhibit viral replication by increasing RT dimer stability, then NNRTI binding pocket mutants that confer drug resistance should decrease the stability of RT heterodimers. Figueiredo *et al.* recently tested this hypothesis by comparing the effects of several mutations in the NNRTI binding pocket on drug resistance and heterodimer stability [95]. They found no obvious correlation between NNRTI resistance and heterodimer stabilization, suggesting that the stability of RT heterodimers is unlikely to be a key player in NNRTI antiviral activity or NNRTI resistance.

An alternative explanation for selection of mutations that reduce RNase H activity in response to NNRTI treatment is that NNRTIs themselves can increase RNase H activity [82,96–99]. It is therefore possible that the C-terminal domain mutations that reduced RNase H activity are selected in response in NNRTI therapy because they restore the balance between RNase H activity and polymerization. Another possible explanation that cannot be excluded is that the cn mutations influence the structure of RT and these structural changes have long-range affects on the NNRTI binding pocket, leading to a reduction in NNRTI binding affinity.

## 7. Prevalence of C-terminal Mutations in Treatment-naïve and Treatment-experienced Patients

**Figure 6:**
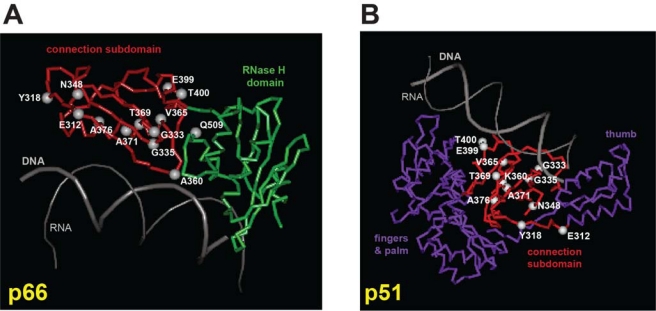
Location of C-terminal domain mutations in p66 **(A)** and p51 **(B)** involved in RTI resistance. Coloring code: red, connection subdomain; green, RNase H domain; purple, fingers and palm and thumb domain; RNA, thin gray line; DNA, thick gray line.

It is important to determine whether the prevalence of specific mutations in the cn and RNase H domains is elevated in patients, and whether the frequency with which these mutations are present is associated with antiviral drug treatment. Relatively small numbers of patients have had their entire RT sequenced; consequently, it is difficult to ascertain the full impact of C-terminal domain mutations on drug treatment, drug resistance, and clinical outcome. Mutations G333D/E and Y318F were the first two cn mutations to be identified to play a role in drug resistance. G333D/E was identified in treatment-experienced patients and shown to confer dual resistance to AZT and 3TC [80,81]; in addition, Y318F, also identified in patients, was shown to enhance DLV resistance by itself, and further enhance NVP and EFV resistance in the presence of classical NNRTI mutations [100,101]. The position of the most commonly identified C-terminal domain mutations in RT is shown in Figure 6 below. A recent publication by Dau *et al.*, examined the overall frequency of these common cn mutations from 345 treatment-experienced patients from the OPTIMA trial [102]. They identified Y318F (4.1%), G333D/E (1.7/13.6%), G335D (5.8%), N348I (12.8%), V365I (7.8%), A371V (21.5%), and A376S (15.7%) to be positively associated with treatment-experienced patients, and as seen in other studies, a positive association of cn mutations with TAMs.

We further analyzed the patient sequences available from the Stanford University Drug Resistance Database (http://hivdb.stanford.edu) to correlate the prevalence of the most common C-terminal domain mutations with the presence of one or more resistance-associated mutations in the pol domain, a surrogate marker for antiviral drug treatment (Table 1). The number of sequences available for analysis ranged from 6035 to 507. The proportion of cn mutations E312Q, Y318F, G333E, N348I, A360V, V365I, T369I, A371V, and A376S was significantly higher for sequences that contained one or more RTI resistance mutations compared to sequences without RTI resistance mutations.

### 7.1. Prevalence of cn Mutations in Patient Databases

N348I has been extensively analyzed in numerous patient cohorts. Yap *et al.*, showed that N348I was highly prevalent (12%) amongst their Canadian patient cohort (n = 1009) and was highly associated with TAMs and NNRTI mutations K103N and Y181C [54]. Hachiya *et al.*, also examined N348I in 48 treatment-experienced clinical isolates from Japan and found that N348I was prevalent in AZT and/or ddI therapy (12.5%), and was also associated with TAMs [103]. Both studies showed that N348I was dual resistant to NRTIs (AZT) and NNRTIs (NVP) *in vitro*. Ehteshami *et al.* examined the prevalence of cn mutations in a Canadian cohort (n = 2422) revealing that in addition to N348I (12.1%), changes in the following cn amino acid positions also increased in prevalence amongst their treatment-experienced patients: 356 (27.2%), 358 (10.9%), 359 (16.4%), 360 (28.8%), 371 (12.7%) and 386 (18%), with A360V also highly associated with classical TAMs [58]. Waters *et al.* in 2009 analyzed 248 treatment-experienced patients and found N348I to be prevalent at a frequency of 24.5% [104], while recently, Price, *et al.*, examined 2,266 treatment-experienced patients from the United Kingdom Collaborative HIV Cohort and found N348I to be present at a frequency 8.7% [105].

Santos *et al.* analyzed 450 sequences from Brazilian subtype B isolates and public databases, and found nine mutations in the cn subdomain (I326V, R358K, G359S, A360T, A360V, K366R, A371V, K390R, and A400T) and six mutations in the rh domain (I506L, K512R, K527N, K530R, and Q547K) that were associated with NRTI drug therapy [106]. Interestingly, only A360V, I506L and Q547K were not found in treatment-naïve patients. Cane *et al.* analyzed over 3000 patients (up to amino acid 400) from a United Kingdom patient database and found that cn subdomain mutations 322, 356, 359, 360, 371 and 381 were associated with the accumulation of TAMs in patients, while cn subdomain mutations 318, 320, 348, 359 and 371 were associated with NNRTI classical resistance mutations [107]. Recently, mutation A400T was also shown to be selected in response NRTIs [108], and Delviks-Frankenberry *et al.* showed that A400T in CRF01_AE was an important determinant for AZT resistance if the patients had acquired TAMs [64]. Furthermore, the Lampang Cohort of CRF01_AE patients failing d4T, 3TC and NVP drug treatment (n = 49) showed that N348I (8%) and E399D (16%) in the cn subdomain (and P537S (5%) and I542M (9%) in the RNase H domain) were associated with treatment failure [109]. The majority of the data collected on drug resistance associated with the C-terminal domain of RT remains limited to subtype B patients. The contribution of C-terminal domain mutations for other subtypes remains to be determined. Analysis of cn subdomains from different subtypes indicates that several positions in the cn subdomain can exhibit polymorphisms. In view of recent appreciation of this diversity, it is important not to mix different subtypes together in cohort studies to examine the frequencies of cn mutations so that prevalence data is not masked.

### 7.2. Prevalence of rh Mutations in Patient Databases

Other cohort studies have focused on mutations localized to the rh domain. In 2007, Roquebert *et al.* analyzed 144 patients from a French cohort for rh mutations, starting at amino acid 427 [110]. A comparison of naïve *vs.* NRTI treatment-experienced patients showed that mutations L469T/I/M/H, T470P/S/E/K, A554T/L/K and K558R/G/E were more prevalent amongst treatment-experienced patients, with K558R/G/E associated with an increase in TAMs. However, Ntemgwa *et al.* analyzed rh mutations in NRTI-experienced patients from a Canadian and an Italian cohort (n = 21) [111] and found positions D460, P468, H483, K512, and S519 to be extensively polymorphic in both treatment-naïve and treatment-experienced patients, but not correlated with high levels of AZT resistance. Even though they found that L469, L491 and K527 were shown to be associated with TAMs, they concluded from their analysis that rh domain mutations were infrequent, found also in naïve patients, and therefore should not be added to routine genotyping. Waters *et al.* in 2009 analyzed 248 treatment-experienced patients and found K451R (11%) from the rh domain to be associated with drug treatment [104]. To date, far fewer mutations in the rh domain have been found to be associated with drug resistance, suggesting that the cn subdomain is likely more tolerable to amino acid changes than the rh domain.

### 7.3. Role of C-terminal Domain Mutations in Clinical Outcome

One large question remains as to whether the presence of C-terminal domain mutations is beneficial or detrimental to the clinical outcome of a patient. Studies on N348I have shown that viral load does increase 0.23 log_10_ copies/ml in the presence of N348I [54], which is similar to that observed with the TAMs mutations alone, suggesting that patients with N348I would have a poorer outcome than patients without C-terminal domain mutations. Hachiya *et al.* has also tried to address this issue by comparing the clinical outcome of patients with and without C-terminal domain mutations who either received or did not receive AZT therapy [112]. They concluded that likely cn and rh domain mutations were acting as pre-therapy polymorphisms and showed that the presence of C-terminal mutations G333E, G333D, V365I, A376S/T/P in their patients did not statistically affect clinical outcome or clinical response regardless of the patients’ AZT therapy status. After 12 weeks on therapy, it does appear that patient viral loads are similar despite whether or not C-terminal domain mutations were present. However, after initiation of therapy, at four and eight weeks, patients who were on AZT therapy and had acquired C-terminal domain mutations, the trend was apparent that these patients had the lowest drop in viral load, suggesting that these patient virus were more drug resistant. Recent data from the OPTIMA trial [102] indicated that presence of any cn mutation did reduce patient virological response (*P* = 0.045), however the authors caution that larger cohorts are needed to definitively answer this question. Overall, it is clear that additional data needs to be extensively collected before any conclusions are made on patient outcomes or clinical relevance of C-terminal domain mutations.

### 7.4. Selection of C-terminal Domain Mutations in HIV-1 Infected Patients

Recent studies have tried to determine how and when cn mutations are selected in HIV-1 infected patients. Soares *et al*. have reported that drug-naïve subjects from Cameroon already contain C-terminal domain mutations; for example, Q509L in one CRF22_01A1 strain and Q547K in one group O strain, suggesting that cn and rh mutations can also potentially play a role in primary drug resistance [113]. Yap *et al.* have shown that N348I is actually selected before the onset of TAMs suggesting that C-terminal domain mutations can play a key role in shaping RTI drug resistance [54] Other studies have tried to link certain drug treatments to the acquisition of cn mutations. Von Wyl *et al.*, examining 50 AZT and 11 3TC monotherapy patients, concluded that *in vivo* selection of N348I is due to AZT drug pressure [63]. Santos *et al.* analyzed patients treated with AZT monotherapy and found a strong association with cn mutations A360V, A371V, K390R and A400T (*P* <.01), suggesting that cn mutations were selected with AZT exposure [106]. Dau *et al.* examined all of the commonly reported cn mutations and found that cn mutations in patients from the OPTIMA trial were associated with ABC, 3TC, d4T, TDF, and AZT treatment; even though the patients had acquired extensive NRTI resistance mutations in pol, the presence of cn mutation(s) further enhanced patient drug resistance [102]. Price *et al.* examined the relationship between N348I and drug treatment in 2,266 treatment-experienced patients and found N348I to be positively associated with EFV and NVP treatment and negatively associated with TDF treatment [105]. This is the first clinical evidence to show that certain drug treatments may also select against a cn mutation. Additionally, mutations T377L and T386I were found to be associated with d4T resistance, and T377L was also found to be associated with ddC resistance in a patient cohort (n = 250) from Italy [114]. Overall, more research is needed to determine how different drug regimens influence the accumulation of C-terminal domain mutations.

## 8. Conclusions

RT is a unique viral protein containing two enzymatic properties: RNase H cleavage activity and RNA- and DNA-dependent DNA polymerase activity. The unique balance between these two activities leads to successful completion of reverse transcription. As shown in this review, mutations in the C-terminal domain of RT that upset this balance can lead to NRTI and NNRTI drug resistance in the HIV-1 infected patient. The C-terminal domain mutations reduce RNase H activity either directly by affecting the RNase H cleavage activity of the enzyme, or indirectly by affecting the overall positioning of the template/primer strand, which in turn affects RNase H activity, template switching, polymerization and/or nucleotide excision. The location of most of these mutations in the cn subdomain and their proximity to the RNase H primer grip residues suggests that they may affect the overall the positioning of the template-primer at the RNase H active site; however, it cannot be ruled out that some of the C-terminal domain mutations may directly affect RNase H cleavage activity. Overall, the data suggests that RNase H function is a unifying mechanism by which C-terminal domain mutations can influence both NRTI and NNRTI resistance.

The extent to which C-terminal domain mutations influence a patient’s clinical outcome is yet to be determined. However, as more and more patient RT sequences are being collected, it is evident that certain C-terminal domain mutations have increased prevalence amongst treatment-experienced patients. Many questions still need to be answered. When do C-terminal domain mutations arise in therapy? Are they influenced by or do they influence the accumulation of other RT mutations? What role do C-terminal domain mutations play in the clinical outcome of the patient? Should the associated increase in NRTI and/or NRTI resistance with C-terminal domain mutations be included when analyzing patient drug regimens? Answers to these questions will hopefully provide valuable information not only for drug resistance and patient therapy regimes, but also future antiviral drug development.

## Figures and Tables

**Table 1:** Prevalence of C-terminal Domain Mutations in RTI Treatment-experienced Patients.

C-terminal domain mutation	No. of sequences containing a C-terminal domain mutation ^a^	No. of sequences containing a C-terminal domain mutation and no RTI ^b^ mutations	No. of sequences containing both a C-terminal domain mutation and ≥1 RTI mutation	Probability of having a C-terminal domain mutation with ≥1 RTI mutation ^c^
**Connection Subdomain**				
E312Q	79/6035 (1.3%)	36/3397 (1.1%)	43/2638 (1.6%)	*P = 0.0350
Y318F	48/5983 (0.8%)	0/3366 (0%)	48/2617 (1.8%)	*P < 0.000001
G333D	47/5086 (0.9%)	21/2864 (0.7%)	26/2222 (1.2%)	*P* =0.0717
G333E	446/5086 (8.8%)	221/2864 (7.7%)	225/2222 (10.1%)	*P = 0.0016
G335C	30/4905 (0.9%)	14/2711 (0.5%)	16/2194 (0.7%)	*P* = 0.2211
G335D	79/4905 (1.6%)	37/2711 (1.4%)	42/2194 (1.9%)	*P* = 0.0802
N348I	180/3189 (5.6%)	5/1213 (0.4%)	175/1976 (8.9%)	*P < 0.000001
A360V	128/3147 (4.1%)	17/1203 (1.4%)	111/1944 (5.7%)	*P < 0.000001
V365I	169/3140 (5.4%)	36/1202 (3.0%)	133/1938 (6.9%)	*P = 0.000001
T369I	19/3115 (0.6%)	1/1195 (0.08%)	18/1920 (0.9%)	*P = 0.0013
A371V	518/3112 (16.6%)	47/1194 (3.9%)	471/1918 (24.6%)	*P < 0.000001
A376S	320/3111 (10.3%)	87/1194 (7.2%)	233/1917 (12.2%)	*P = 0.000006
E399D	475/2968 (16%)	178/1072 (16.6%)	297/1896 (15.7%)	*P* = 0.7656
A400T	455/1616 (28.2%)	205/628 (32.6%)	250/988 (25.3%)	*P* = 1.00
**RNase H Domain**				
Q509L	2/507 (0.4%)	2/304 (0.7%)	0/203 (0%)	*P* = 1.00

a Data from the Stanford HIV Drug Resistance Database, Detailed RT Mutation Profile Program, as of April 2010.

b Major and minor RTI mutations were those defined by the Stanford database. Major NRTI mutations (http://hivdb.stanford.edu/pages/documentPage/NRTI_mutationClassification.html) included 41L, 65R/N, 67N, deletion D67, insertion at T69, 69D, 70R/E/G, 74I/V, 75T/A/M, 115F, 151M/L, 184V/I, 210W, and 215Y/F. Minor NRTI mutations included 41 not L, 44D/A, 62V, 67 not deletion or N, 69 not insertion or D, 70 not R/E/G, 74 not I/V, 75 not T/A/M, 77L, 115 not F, 116Y, 118I, 151 not M/L, 184 not V/I, 210 not W, 215 not Y/F, 219 Q/E/N/R/W, 333D/E, and 348I. Major NNRTI mutations (http://hivdb.stanford.edu/pages/documentPage/NNRTI_mutationClassification.html) included 100I, 101E/P, 103N/S/T/H, 106A/M, 179F, 181C/I/V, 188C/H/L, 190A/S/E/Q/T/C/V, 230L, and 236L. Minor NNRTI mutations included 90I, 98G, 100 not I, 101 Q/H/N, 103 not N/S/T/H, 106 not A/M/I/L, 108I, 138K, 179D/E, 181 not C/I/V, 188 not C/H/L, 190 not A/S/E/Q/T/C/V, 225H, 227C/L, 234I, 236 not L, 238N/T, 318F and 348I. The word “not” refers to all mutations at that position except the following mutation(s).

c Two proportions statistics were performed by comparing the number of C-terminal domain mutations with at least one RTI mutation (for example, 43 for E312Q) to the total number of sequences containing at least one RTI mutation (2638 of 6035), against the number of C-terminal domain mutations without an RTI mutation (for example, 36 for E312Q) to the total number of sequences without an RTI mutation (3397 of 6035).
